# Plasma profile and urine excretion of amino acids in children with celiac disease on gluten-free diet after oligofructose-enriched inulin intervention: results of a randomised placebo-controlled pilot study

**DOI:** 10.1007/s00726-018-2622-7

**Published:** 2018-07-24

**Authors:** Natalia Drabińska, Urszula Krupa-Kozak, Ewa Ciska, Elżbieta Jarocka-Cyrta

**Affiliations:** 1 Department of Chemistry and Biodynamics of Food, Institute of Animal Reproduction and Food Research of Polish Academy of Sciences, Tuwima 10 Str., 10-748 Olsztyn, Poland; 2 Department of Pediatrics, Gastroenterology and Nutrition, Collegium Medicum, University of Warmia & Mazury, Oczapowskiego 2, Str., 10-719 Olsztyn, Poland

**Keywords:** Amino acids, Celiac disease, Oligofructose-enriched inulin, Dietary intervention, Clinical trial, Gluten-free diet

## Abstract

The circulating amino acid (AAs) concentrations are indicators of dietary protein intake and metabolic status. In celiac disease (CD), the AA imbalance is frequently observed. Prebiotics are found to alleviate nutrient deficiencies. Therefore, the aim of this study was to analyse the impact of oligrofructose-enriched inulin (Synergy 1), administered for 3 months as a gluten-free diet (GFD) supplement to children with CD, on the plasma and urine concentrations of AAs. CD children (*N* = 34) were randomised into two groups, receiving Synergy 1 (10 g/day) or placebo (maltodextrin) for 3 months. The AA profile and concentration was determined in plasma and urine before and after the dietary intervention by gas chromatography. 22 and 28 AAs were determined in plasma and urine samples, respectively. After the intervention, the plasma concentrations of several AAs (Ala, Pro, Asn, Glu, Tyr, Lys, His, Orn) increased significantly in both experimental groups, while Gln increased only in the Synergy 1 group. The urinary excretion of Asn, Lys and Aaa increased significantly in the Synergy 1 group, and the excretion of Asp and Met decreased (*p* < 0.05) in the placebo group. The Gln:Glu ratio in urine increased in both groups after the intervention. An increased urinary excretion of AAs observed in Synergy 1 group with a simultaneous increase in the content of circulating AAs could be attributed to higher absorption or intensified metabolism of AAs, and on the other hand further healing of the intestinal mucosa being the result of continuous treatment with GFD. Moreover, the observed changes in Glu concentration suggest that oligofructose-enriched inulin could improve the intestinal condition and permeability. To conclude, a prebiotic-supplemented GFD influences beneficially the overall AAs metabolism in CD children; however, further prospective cohort studies are needed to confirm the results obtained.

## Introduction

Chronic intestinal inflammation alters gut functions by weakening the intestinal barrier, damaging epithelial cells and decreasing absorption (Barker and Liu 2008), which deplete the levels of important nutrients and mediators in the human body, including amino acids (AAs). AAs are essential metabolites which play a crucial role in the human body. They participate in the formation of protein structure, regulation of anabolic and catabolic metabolism, and detoxification processes. Dietary AAs act as substrates for the development of epithelial cells and mucin synthesis, they maintain the integrity of the gut barrier and play a pivotal role in the intestinal immune system (Fillmann et al. 2007; Ren et al. 2014; Zhang et al. 2015). AAs are the precursors for the synthesis of important mediators, such as glutathione, an antioxidant with cytoprotective activity which prevents epithelial cell damage (Wu et al. 2004). The concentrations of circulating AAs are reliable indicators of dietary protein intake and metabolic status. Recent research has focused on the AA balance and changes in AA metabolism as factors that contribute to the development of pathological conditions. Changes in the AA profile often accompany cancerogenesis, mental disorders and fatigue (Evans et al. 2008; Hugh Dunstan et al. 2011; Dereziński et al. 2017). The AA imbalance is also frequently observed in celiac disease (CD) (Di Cagno et al. 2011; Sevinc et al. 2015).

CD is a chronic autoimmune enteropathy which is triggered by gluten ingestion and is observed in genetically predisposed individuals. The only treatment for CD is a gluten-free diet (GFD) which may be difficult to optimise in terms of fulfilling the patients’ nutritional needs (Roma et al. 2010; Ilus et al. 2014). CD patients who have been following a GFD for a long time are characterised by sub-optimal levels of essential AAs (EAAs) in biological fluids, compared with healthy individuals (van Hees et al. 2015). The psychological symptoms that accompany CD, such as anxiety, irritability, depression and apathy (Carta et al. 2002; Addolorato et al. 2008), are linked with a reduced intake of dietary tryptophan and, consequently, decreased synthesis of monoamines, including serotonin (Hernanz and Polanco 1991). According to many metabolic studies (Bertini et al. 2009; Di Cagno et al. 2009), the AA balance plays a very important role in CD management. Despite the above, AA profiling in CD has been scarcely researched (Sevinc et al. 2015).

According to the literature, prebiotics could be helpful in alleviating nutrient deficiencies by regulating the composition and the activity of gut microbiota. In vitro and in vivo studies as well as clinical trials involving patients with intestinal inflammatory diseases have demonstrated that inulin-type fructans improve mineral absorption, alleviate the symptoms of inflammation and induce morphological changes in the intestines (Guarner 2007; Krupa-Kozak et al. 2016; Shoaib et al. 2016). The applicability of inulin-type fructans in GFD and its effects on the AA profile in biological fluids have not been investigated to date. Therefore, the aim of this study was to analyse the impact of oligrofructose-enriched inulin, administered for 3 months as a GFD supplement to children with CD, on the plasma and urine concentrations of selected AAs.

## Materials and methods

### Experimental design

A randomised, placebo-controlled, parallel-group and single-centre pilot study was performed on children and adolescents (*N *= 34, aged from 4 to 18 years) diagnosed with CD based on the criteria of the European Society for Paediatric Gastroenterology, Hepatology and Nutrition (ESPGHAN) (Husby et al. 2012), and following GFD since at least 6 months (GFD adherence in a range from 6 months to 9 years) to assess the effects of oligofructose-enriched inulin (Synergy 1; Orafti^®^ Synergy 1, Beneo, Tienen, Belgium) on their health and nutritional status. All participants met the inclusion criteria: CD confirmed by serological, genetic and biopsy analyses; GFD for at least 6 months. The exclusion criteria included: use of antibiotics in the month preceding the study; use of medication for osteoporosis (bisphosphonates, calcium calcitonin); use of probiotics, prebiotics or fibre supplements; poor or average overall health; current enrolment in another research study; recent surgery. The detailed protocol of the clinical trial, and the demographic and anthropometric characteristics of the participants at the beginning of the study have been described previously (Krupa-Kozak et al. 2017). The patients were randomised into two groups: based on sex and age: the Synergy 1 group (*N* = 18; girls = 11; age 5–17 years, average 10 years) and the placebo group (*N* = 16; girls = 10; age 4–16 years, average 10 years). Over a period of 3 months, the participants in the Synergy 1 group were administered oligofructose-enriched inulin at 10 g/day, and the participants in the placebo group were administered maltodextrin at 7 g/day. Patients of both groups continued to follow a strict GFD throughout the whole experiment. Among recruited subjects, 30 completed the study and were included in the final analysis, while four children were excluded, in particular two subjects who had been treated with antibiotics during the experiment and two subjects who failed to comply with the experimental requirements.

Anti-tissue transglutaminase antibody (tTG) assay has been performed before and after the intervention as a part of clinical practice by Hospital’s Diagnostic Laboratory to control adherence to GFD and/or progress of the treatment. The results of tTG assay are presented in Table 1.

**Table 1:** Values of anti-tissue transglutaminase in CD children before and after the intervention, expressed as a median (P25–P75). Normal range is < 8.0 AU/mL

	T0		T1	
	Synergy 1	Placebo	Synergy 1	Placebo
tTG [AU/mL]	2.57 (0.48–6.98)	2.17 (0.85–3.74)	1.22 (0.34–4.88)	1.49 (0.62–4.83)

### Sample collection

Urine and blood samples were collected from each participant at baseline and after the 3-month dietary intervention. For ethical reasons, samples were collected during regular follow-up visits (every 3 months) in the Gastroenterology Clinic of the Children’s Hospital in Olsztyn (Poland). Blood samples were collected into vacuum tubes containing an anticoagulant (heparin). Plasma was divided into aliquots (100 μL) and stored at − 80 °C until AA analyses. Approximately 50 mL of morning urine (second spontaneous urine) was collected, divided into aliquots and stored at − 80 °C until further analysis.

### Amino acid analysis

The AA profile in urine and plasma samples was analysed using the EZ:Faast™ Kit for Free (Physiological) Amino Acids (Phenomenex, Aschaffenburg, Germany) according to the producer’s recommendations. The chromatographic method was chosen due to its ability to detect a wide range of AAs in a single run and in a very short time (7 min) using a relatively small amount of the sample. The analytical procedure involves solid-phase extraction of 100 μL of urine or plasma, followed by derivatization and liquid–liquid extraction. AAs were identified in the Agilent 7890A gas chromatograph (Agilent Technologies, Santa Clara, CA, USA) coupled with the 5975C mass selective detector, 7683B auto-injector and a data station containing the NIST/EPA/NIH Mass Spectral Library (Version 2). The compounds were separated in the ZB-AAA EZ Faast™ capillary column (10 m × 0.25 mm). The carrier gas was helium (1.5 mL/min). The samples (2 µL) were injected in split mode (1:15). Oven temperature was initially set at 110 °C and then increased to 320 °C (30 °C/min). Injector and MS source temperatures were 250 and 240 °C, respectively. Mass spectra were obtained by electron ionisation (EI) over the range of 35–550 m/e. Electronic impact energy was 70 eV. Amino acids were identified using calibration standards for each AA, and a quantitative analysis was performed relative to the internal standard (norvaline).

### Statistical analysis

All analyses were performed in duplicates. Raw data were expressed as means ± SEM. The Gln:Glu ratio was presented as means ± SD. Data were processed in Statistica 12 software (StatSoft, USA). Only the data collected from participants whose reported intake of the supplements exceeded 80% were included in the final analysis. Normal distribution was evaluated in the Shapiro–Wilk test. Analyses comparing both groups at baseline and after 3 months were performed separately using the Student’s *T* test or the Mann–Whitney *U* test as appropriate. Comparison within groups between baseline and after 3 months was performed using the paired samples Student’s *T* test or Wilcoxon test, as appropriate. Statistical significance thresholds were set at *p* < 0.05 (^*^) and *p* < 0.01 (^**^).

## Results

### Amino acid profile in plasma

Twenty-two AAs, including eight non-essential AAs (NEAAs) (Ala, Gly, Ser, Pro, Asn, Gln, Glu, Tyr), nine EAAs (Val, Leu, Ile, Thr, Met, Phe, Lys, Trp, His) and five non-proteinogenic AAs and derivatives (Aba, Orn, Hyp, C–C, Php), were determined in plasma samples collected from children with CD (Table 2). At baseline, the plasma profile and the concentrations of individual AAs and total AA in did not differ between the Synergy 1 group and the placebo group.

**Table 2:** Concentrations of AAs and derivatives in the plasma of children with celiac disease (*N* = 30): a comparison of the group supplemented with Synergy 1 and the placebo group at baseline and after 3 months of supplementation

		T0				T1				S-T0 vs. S-T1	P-T0 vs. P-T1	S-T1 vs. P-T1
		Synergy 1 (S)		Placebo (P)		S		P				
		Av	SEM	Av	SEM	Av	SEM	Av	SEM			
Non-essential amino acids												
Alanine	Ala	284.70	21.73	280.96	20.76	387.94	22.48	406.25	30.91	0.008**	0.012*	0.629
Glycine	Gly	226.80	20.58	210.69	16.33	248.97	16.50	229.08	16.97	0.110	0.286	0.325
Serine	Ser	109.19	7.72	93.40	5.65	123.91	10.26	119.36	8.65	0.196	0.008**	1.000
Proline	Pro	137.27	6.48	129.46	9.59	164.65	9.57	199.39	19.38	0.028*	0.007**	0.197
Asparagine	Asn	46.89	3.28	43.62	3.30	54.52	2.70	57.89	2.72	0.006**	0.008*	0.395
Glutamine	Gln	457.39	25.48	411.34	18.35	498.93	16.11	473.37	36.35	0.015*	0.092	0.485
Glutamic acid	Glu	22.86	2.65	25.69	2.85	48.63	2.43	39.41	2.86	0.007**	0.043*	0.037*
Tyrosine	Tyr	43.67	2.64	41.01	2.40	54.19	3.84	61.14	6.06	0.030*	0.016*	0.433
Essential amino acids												
Valine	Val	225.65	18.04	202.43	15.13	241.97	11.91	244.09	24.88	0.743	0.145	0.936
Leucine	Leu	101.49	7.79	85.91	4.01	110.78	8.87	124.23	15.55	0.182	0.008**	0.761
Isoleucine	Ile	51.36	3.33	45.51	2.33	53.29	1.98	57.34	4.50	0.541	0.084	0.363
Threonine	Thr	110.59	7.94	105.11	11.01	136.35	9.31	137.46	9.78	0.112	0.047*	0.935
Methionine	Met	11.53	0.88	10.02	0.66	13.52	0.65	14.65	1.72	0.071	0.012*	0.483
Phenylalanine	Phe	35.81	1.70	35.35	1.64	39.15	2.50	41.20	1.98	0.186	0.034*	0.542
Lysine	Lys	193.74	8.27	182.73	6.81	247.26	10.06	267.94	14.36	0.011*	0.007**	0.240
Tryptophan	Trp	43.23	2.49	39.21	1.89	46.30	3.46	55.39	4.43	0.051	0.018*	0.070
Histidine	His	65.28	3.12	61.36	3.65	75.16	3.11	86.36	6.72	0.042*	0.016*	0.218
Other amino acids and derivatives												
α-Aminobutyric acid	Aba	17.07	1.27	15.43	1.11	17.88	1.44	19.36	1.75	0.421	0.011*	0.685
Ornithine	Orn	47.85	2.14	42.45	2.79	59.71	2.48	72.95	7.69	0.012*	0.002*	0.174
4-hydroxyproline	Hyp	20.03	2.78	18.79	2.22	22.07	1.88	27.14	3.04	0.955	0.008**	0.164
Cystine	C–C	25.94	3.81	23.89	3.34	25.08	3.22	35.39	4.31	0.953	0.028*	0.193
Proline–hydroxyproline	Php	9.44	1.27	6.89	1.30	8.42	2.64	10.82	3.22	0.463	0.089	1.000

Data are given as the mean with SEM in nmol/mL. Differences were considered significant as follow: (*) *p* < 0.05; (**) *p* < 0.01

**Fig. 1:**
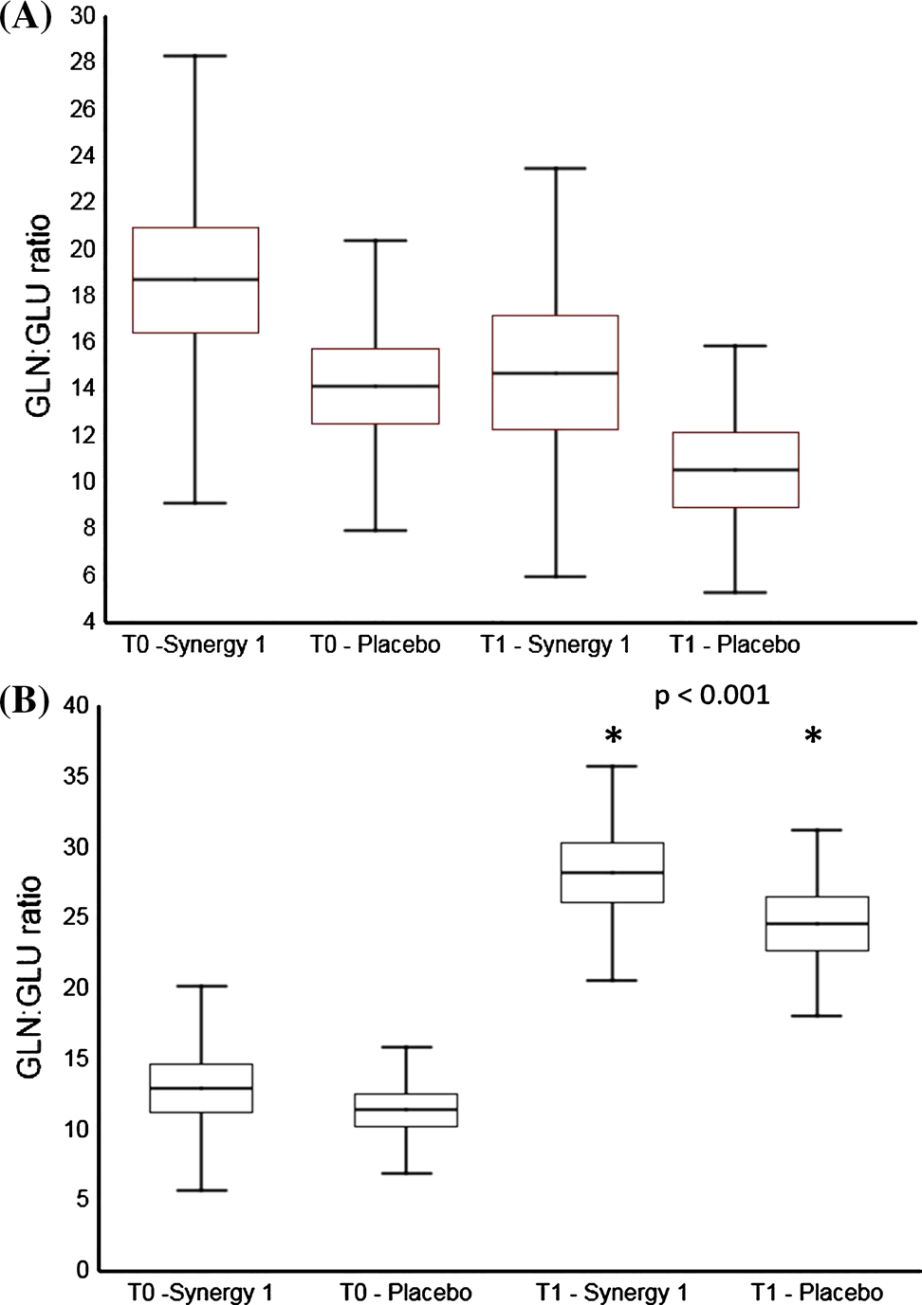
Box-and-whisker plots of the Gln:Glu ratio in the plasma **a** and urine **b** of children with celiac disease: a comparison of the group supplemented with Synergy 1 and the placebo group at baseline and after 3 months of supplementation; Mann–Whitney test

**Fig. 2:**
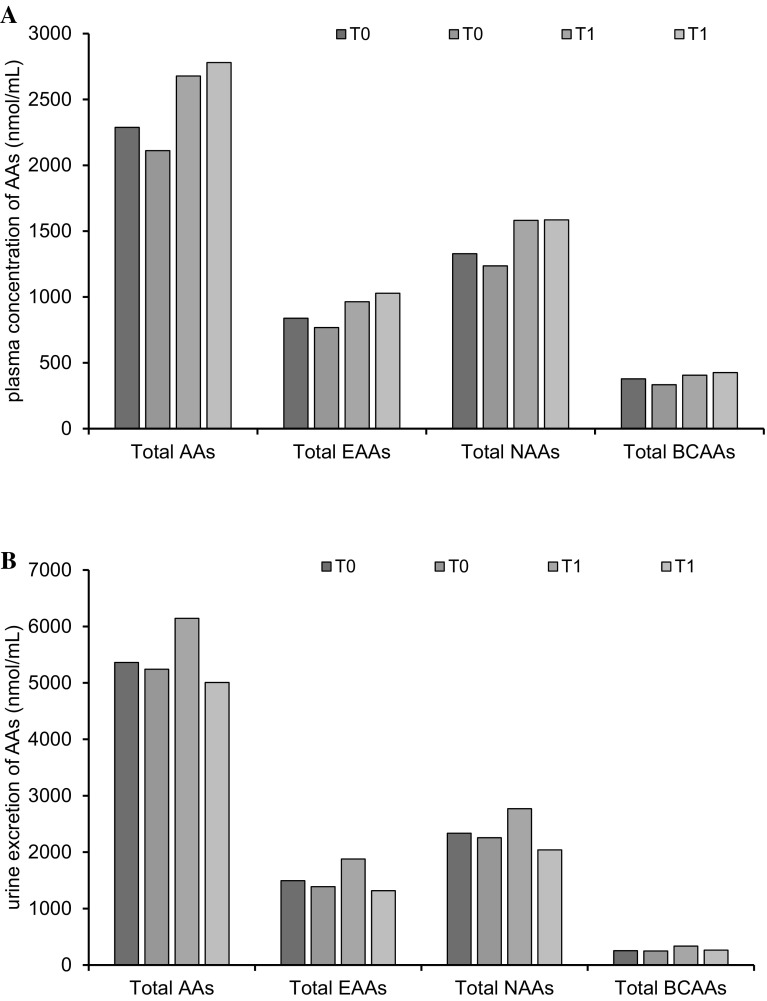
Summarized values of total concentrations of all, essential (EAAs), non-essential (NEAAs) and branched-chain (BCAAs) amino acids in plasma **a** and urine **b** of children with CD from Synergy 1 and placebo group, before (T0) and after (T1) the intervention

After the 3-month dietary intervention, the concentrations of NEAAs, in particular Ala, Pro, Asn, Glu, Tyr, as well as Lys, His and Orn increased significantly (*p* < 0.05) in both groups, regardless of the applied GFD supplementation (Table 2). The concentration of Gln increased significantly (*p* < 0.05) only in Synergy 1 group. On the other hand, a significant increase of the concentration of majority of EAAs and of Aba, Hyp and C–C in plasma was observed only in the placebo group. Interestingly, the administration of Synergy 1 significantly increased (*p* < 0.01) the plasma Glu level, but did not affect the Gln:Glu ratio which remained similar to baseline (Fig. 1a). Consequently, the total AA concentration increased by 17% in the Synergy 1 group and by 32% in the placebo group relative to baseline (Fig. 2a). The total concentration of EAAs increased by 15% in the Synergy 1 group and by 23% in the placebo group, and the total concentration of NEAAs increased by 19% in both groups. The total concentration of branched-chain amino acids (BCAAs) increased by 7% in the Synergy 1 group and by 13% in the placebo group.

### Amino acid profile in urine

The urinary AA profile determined in Synergy 1 and placebo groups at baseline and after the dietary intervention is presented in Table 3. Twenty-eight AAs, including nine NEAAs (Ala, Gly, Ser, Pro, Asn, Asp, Gln, Glu, Tyr), nine EAAs (Val, Leu, Ile, Thr, Met, Phe, Lys, Trp, His) and ten AA derivatives and non-proteinogenic AAs (Aba, βAiB, Aaa, Apa, Orn, Hly, Cth, C–C, Gpr, Php), were identified. The total urinary concentration of AAs in both groups was similar at baseline at approximately 5300 nmol mL^−1^. The predominant AAs were Gly, His and Php. The main AA fractions were NEAAs which accounted for 43% of total AAs, EAAs accounted for approximately 27%, and BCAAs—for 5% of total AAs.

**Table 3:** Urinary excretion of AAs and derivatives in children with celiac disease (*N* = 30): a comparison of the group supplemented with Synergy 1 and the placebo group at baseline and after 3 months of supplementation

		T0				T1				S-T0 vs. S-T1	P-T0 vs. P-T1	S-T1 vs. P-T1
		Synergy 1 (S)		Placebo (P)		S		P				
		Av	SEM	Av	SEM	Av	SEM	Av	SEM			
Non-essential amino acids												
Alanine	Ala	281.19	23.60	255.84	24.19	365.44	35.20	253.49	32.15	0.124	0.852	0.045*
Glycine	Gly	1073.80	120.30	1056.63	170.99	1286.25	174.02	924.67	122.09	0.116	0.753	0.174
Serine	Ser	333.35	24.78	321.13	27.47	403.63	36.02	309.02	34.78	0.124	0.917	0.059
Proline	Pro	9.59	0.51	9.26	0.75	10.18	0.90	8.38	0.90	0.557	0.263	0.170
Asparagine	Asn	118.38	8.40	115.89	16.93	147.43	13.49	101.52	14.48	0.034*	0.499	0.018*
Aspartic acid	Asp	9.09	0.98	11.80	1.25	9.11	0.70	8.82	0.80	0.750	0.028*	0.836
Glutamine	Gln	378.13	30.79	361.13	47.15	425.37	25.67	329.79	22.46	0.088	0.128	0.015*
Glutamic acid	Glu	35.79	5.00	32.14	5.08	17.36	1.48	13.32	1.21	0.006**	0.017*	0.094
Tyrosine	Tyr	95.65	6.92	91.15	10.50	105.41	15.06	91.88	11.57	0.575	0.799	0.193
Essential amino acids												
Valine	Val	43.98	4.51	40.29	4.46	46.02	5.84	39.14	3.58	0.517	0.810	0.369
Leucine	Leu	33.42	2.54	31.43	2.80	37.64	5.90	30.62	3.63	0.310	0.866	0.153
Isoleucine	Ile	15.91	1.71	15.09	1.87	16.43	2.01	13.54	1.57	0.515	0.214	0.145
Threonine	Thr	121.01	9.87	116.83	14.40	149.31	17.63	114.96	18.08	0.240	0.767	0.142
Methionine	Met	16.74	2.66	16.80	2.90	16.80	3.48	13.18	1.76	0.794	0.043*	0.306
Phenylalanine	Phe	50.71	4.36	46.42	5.57	59.79	7.31	43.11	4.51	0.532	0.678	0.045*
Lysine	Lys	183.45	21.01	176.96	10.80	252.87	32.66	202.58	33.39	0.017*	0.273	0.116
Tryptophan	Trp	60.43	4.25	65.20	8.17	71.08	9.41	64.43	7.32	0.646	0.859	0.278
Histidine	His	968.44	82.05	879.50	73.38	1228.07	196.18	795.39	112.43	0.273	0.893	0.068
Other amino acids and derivatives												
α-Aminobutyric acid	Aba	16.05	1.34	16.52	1.91	16.68	1.47	16.18	1.67	0.253	0.575	0.369
β-Aminoisobutyric acid	Baib	160.46	32.45	161.88	39.61	234.65	32.48	181.01	33.22	0.116	0.180	0.393
α-Aminoadipic acid	Aaa	35.99	4.05	32.41	5.08	42.18	3.51	37.95	5.96	0.041*	0.128	0.203
α-Aminopimelic acid	Apa	12.87	2.14	15.38	2.36	17.01	2.01	10.42	0.39	0.075	0.110	0.013*
Ornithine	Orn	33.35	3.95	34.69	4.68	35.41	3.60	31.15	3.69	0.155	0.779	0.163
Hydroxylysine	Hly	83.06	9.92	83.44	10.91	102.38	10.26	81.66	8.07	0.310	0.499	0.081
Cystathionine	Cth	109.91	10.50	118.54	13.04	124.39	26.99	147.71	17.06	0.753	0.310	0.597
Cystine	C–C	65.88	7.53	59.93	7.59	88.86	11.85	64.18	7.73	0.514	0.889	0.142
Glycyl-proline	Gpr	97.52	9.75	126.11	14.12	124.93	13.97	129.04	16.56	0.116	0.735	0.971
Proline–hydroxyproline	Php	917.24	92.63	949.76	101.12	708.86	63.85	949.32	105.60	0.225	0.917	0.597

Data are given as the mean with SEM in nmol/mL. Differences were considered significant as follows: (*) *p* < 0.05; (**) *p* < 0.01

At baseline, the urine profile and the concentrations of individual AAs and total AA did not differ between the experimental groups, whereas noteworthy changes in the urinary excretion of individual AAs were observed after the dietary intervention (Table 3). The urinary excretion of Asn, Lys and Aaa increased significantly (*p* < 0.05) in the Synergy 1 group, and the excretion of Asp and Met decreased (*p* < 0.05) in the placebo group relative to baseline values. Interestingly, the urinary concentration of Glu in both experimental groups decreased by half independently on applied supplement. An analysis of individual AA concentrations in urine after the intervention revealed higher excretion of Ala, Asn, Gln, Phe and Apa in the prebiotic group than in the placebo group. Consequently, urinary excretion of total AAs, EAAs and NEAAs increased by more than 14, 25 and 18%, respectively, in the Synergy 1 group, whereas a decrease in total AA concentrations was observed in the placebo group (Fig. 2b). In the Synergy 1 group, urinary excretion of BCAAs increased by 32% from baseline and was higher than in the placebo group where only a 4% increase was noted. The urinary Gln:Glu ratio was calculated for individual patients in each group, and the results are presented in Fig. 1b. The Gln:Glu ratio increased significantly in both groups after the intervention, compared with the baseline value.

## Discussion

The disturbances in the intestinal microbiota, mainly lower number and variety of *Bifidobacterium* sp. being capable of digesting immunogenic gliadin peptides, have great importance in CD pathophysiology and affect the metabolic profiles in biological fluids of CD patients (Cukrowska et al. 2017). In the present study, we analysed the impact of dietary intervention with prebiotic applied as a supplement of a GFD on the profile and the concentration of AAs in the plasma of children with CD. This is the first ever study to analyse plasma AA levels with the use of the EZ:Faast™ method; therefore, our results cannot be reliably compared with the findings of other authors. To data, only few studies have investigated the AA profile in the urine or plasma of CD patients. Sevinc et al. (Sevinc et al. 2015) noted a lower levels of Gln and C–C and significantly higher concentrations of Ala, Asn, Glu, Hyp, Ile, Leu, Phe, Pro, Ser, Thr and Val in the plasma of children with CD children than in healthy subjects. The analysis of urinary concentrations of AAs in CD patients showed lower levels of Glu and Gln and higher excretion of Gly compared to healthy controls (Bertini et al. 2009). Children with CD who followed a GFD were characterised by higher concentrations of Lys and Arg and lower Gln levels in urine, compared with healthy controls (Di Cagno et al. 2011). The observed variations in the concentrations of individual AAs between health and CD patients could be attributed to differences in dietary patterns, intestinal malabsorption and inflammatory processes (Sevinc et al. 2015; van Hees et al. 2015).

In general, we detected that after the 3-month dietary intervention, regardless the administered supplement, the total plasma concentration of AAs increased in children with CD. The above result could suggest that a strict GFD alone delivers benefits. The total concentrations of EAAs and NEAAs increased in both groups, which suggest that the continuation of GFD over a period of 3 months induced positive changes in the intestines. The scientific data indicated that the strict adherence to a GFD has beneficial effect on CD patients by reducing symptoms and health care consumption (Norström et al. 2012); therefore, it can be suggested a continuous healing of the intestines in all patients could be related to a good adherence to GFD (Table 1). In the literature, a decrease in the concentrations of the above EAAs has been related to severe intestinal failure, and those EAAs as well as citrulline are regarded as markers of mucosal damage (Fragkos et al. 2015). Interestingly, the plasma concentrations of selected AAs, including Ser, Leu, Thr, Met, Phe, Trp, Aba, Hyp and C–C, increased only in the placebo group. The observed differences in plasma AA levels between groups could be attributed to the specific properties of the administered prebiotic. Prebiotics are substrates which are utilised by gut microbiota during the formation of short-chain fatty acids (SCFAs). Davila et al. (Davila et al. 2013) suggested that AAs could be used by prebiotic-activated gut microbiota to synthesise SCFAs. Gly, Ala, Thr, Glu, Lys and Asp are the precursors of acetate, whereas butyrate is derived from Glu and Lys, and propionate is synthesised from Ala and Thr (Davila et al. 2013). In our previous study, SCFA levels increased in children with CD after the administration of Synergy 1 (Drabińska et al. 2018). Therefore, lower levels of some individual AAs in the Synergy 1 group compared to the placebo group could suggest that these AAs are transformed in the metabolic pathway and/or are utilised for the production of SCFAs.

In the present study, we noted that the supplementation of GFD with Synergy 1 increased the total AA concentration in urine. Increased urinary excretion of AAs with a simultaneous increase in the content of circulating AAs could be attributed to higher absorption and intensified metabolism of AAs. Therefore, the simultaneous analysis of AAs in urine and plasma seems to be reasonable to avoid misinterpretation. On the other hand, higher urinary excretion of AAs could be associated with the loss of AA caused by metabolic failure, including intestinal permeability and failure in the kidney reabsorption processes (Dunstan et al. 2017). Urinary excretion of AAs decreases in consequence of biochemical and metabolic disorders (Evans et al. 2008) or impaired protein synthesis (Niblett et al. 2007). In a group of autistic patients following a gluten-free and casein-free diet, urinary excretion of AAs increased to a level approximating that noted in healthy controls (Evans et al. 2008). Similar tendency was observed in our study, suggesting positive changes in the metabolism.

Positive changes in Glu concentration were noted after the conducted dietary intervention. In plasma, the concentration of Glu nearly doubled in the Synergy 1 group relative to baseline values, whereas only a 50% increase was noted in the placebo group. Moreover, urinary excretion of Glu decreased in both groups. Glu is a key factor responsible for the maintenance of gut function. Plasma Glu levels are relatively low because this AA is the main substrate for intestinal epithelial cells and a precursor in the synthesis of other AAs (Ala, Aap, Orn, Pro) and mediators (glutathione) (Ruth and Field 2013). There is evidence to indicate that Glu plays an important role in the intestinal barrier function. The supplementation of animal diets with Glu changed intestinal morphology, increased villus height and mucosal thickness in the jejunum (Wu et al. 2012). In the Synergy 1 group, the concentration of Gln, a precursor in Glu synthesis, was characterised by a minor increase in plasma samples and a significant increase in urine samples relative to the placebo group. Gln also plays an important role in the gut function, participates in the synthesis of other AAs and protein, influences redox homeostasis and the immune response (Wang et al. 2009; Ruth and Field 2013). Numerous in vitro and in vivo studies have shown that Gln plays a pivotal role in the maintenance of intestinal integrity (DeMarco et al. 2003; Nose et al. 2010). According to Guarner (Guarner 2007), prebiotics contribute to the preservation of gut barrier integrity. Inulin-enriched diets improved intestinal architecture in rats and mice (Kleessen et al. 2003; Strugala et al. 2003; Liu et al. 2016). In most randomised human trials, inulin had no effect on intestinal permeability in patients with burn injuries, critically ill patients or healthy individuals (Jain et al. 2004; Olguin et al. 2005; Ten Bruggencate et al. 2006). Despite the above, Russo et al. observed that inulin modified the concentrations of two intestinal permeability markers, zonulin and glucagon-like peptide 2, and suggested that inulin could be used in the prevention of intestinal disorders (Russo et al. 2012). Further research is needed to explore the ambiguous influence of prebiotics on intestinal barrier function.

The Gln:Glu ratio plays an important role in nitrogen metabolism, brain functions and the gut balance (Hashimoto et al. 2005; Abu Shmais et al. 2012). In the present study, the Gln:Glu ratio was determined in both urine and plasma. Significant changes in Gln:Glu ratio were not observed in plasma, but the analysed parameter increased significantly in urine in both groups after the intervention. These findings could suggest that GFD on its own induces beneficial changes in the intestines. A recent study of patients with chronic intestinal pseudo-obstruction demonstrated a significant decrease (*p* < 0.001) in the urinary Gln:Glu ratio in patients diagnosed with intestinal failure in comparison with healthy controls (Yan et al. 2017).

The present pilot study has certain limitations. The number of participants was low, and the size of the sample and the power of the study could not be determined due to the lack of information from previous studies. However, these limitations are generally associated with pilot studies, and the obtained data will be used to calculate the necessary sample size in future studies. Due to the limited number of participants, a large-scale study should be performed to confirm the results obtained in this pilot study. Last, the present trial was conducted on children whose metabolism differs from that of adults. Therefore, the following study should be performed on older patients to generalise the findings of this research.

## Conclusions

The present randomised placebo-controlled study makes a pioneering attempt to evaluate the influence of a dietary prebiotic on the AA profile of children diagnosed with CD and following a GFD. Our results indicate that prebiotic-supplemented GFD impact the overall AAs metabolism in children with CD, determined in two types of bodily fluids, plasma and urine. In group supplemented with Synergy 1, an increased urinary excretion of AAs with a simultaneous increase in the content of circulating AAs was detected that could be attributed to higher absorption and/or intensified metabolism of AAs, as well as further healing of the intestinal mucosa. The observed changes in Glu concentration suggest that oligofructose-enriched inulin could improve the intestinal condition and permeability. Further research is needed to explore the effect of oligofructose-enriched inulin on intestinal permeability.
